# Vascular and Neuronal Protection in the Developing Retina: Potential Therapeutic Targets for Retinopathy of Prematurity

**DOI:** 10.3390/ijms20174321

**Published:** 2019-09-03

**Authors:** Jessica K. W. Tsang, Jin Liu, Amy C. Y. Lo

**Affiliations:** Department of Ophthalmology, Li Ka Shing Faculty of Medicine, The University of Hong Kong, Hong Kong, China

**Keywords:** oxygen-induced retinopathy, neovascularization, vascular protection, vascular endothelial growth factor, animal models, supplementary oxygen therapy, neuron, eye

## Abstract

Retinopathy of prematurity (ROP) is a common retinal disease in preterm babies. To prolong the lives of preterm babies, high oxygen is provided to mimic the oxygen level in the intrauterine environment for postnatal organ development. However, hyperoxia-hypoxia induced pathological events occur when babies return to room air, leading to ROP with neuronal degeneration and vascular abnormality that affects retinal functions. With advances in neonatal intensive care, it is no longer uncommon for increased survival of very-low-birth-weight preterm infants, which, therefore, increased the incidence of ROP. ROP is now a major cause of preventable childhood blindness worldwide. Current proven treatment for ROP is limited to invasive retinal ablation, inherently destructive to the retina. The lack of pharmacological treatment for ROP creates a great need for effective and safe therapies in these developing infants. Therefore, it is essential to identify potential therapeutic agents that may have positive ROP outcomes, especially in preserving retinal functions. This review gives an overview of various agents in their efficacy in reducing retinal damages in cell culture tests, animal experiments and clinical studies. New perspectives along the neuroprotective pathways in the developing retina are also reviewed.

## 1. Introduction

Retinopathy of prematurity (ROP) is a vasoproliferative retinal disease in the preterm babies. It was initially described and named as retrolental fibroplasia in 1942 [1]. In the normal human fetus with a gestational period of 40 weeks, retinal vascular development starts at gestational week 16, proceeding from the center toward the peripheral retina. In the full-term babies, the retina, including the vessels and neuronal cells, is well-developed at birth, but not in preterm infants. As the preterm infant has an immature cardiopulmonary system, it needs to be placed in high supplemental oxygen. This places the development of the retina at risk and results in ROP.

With the advances in neonatal intensive care, it is no longer uncommon for the increased survival of very-low-birth-weight preterm infants, therefore, increasing the incidence of ROP. Indeed, ROP is now a major cause of preventable childhood blindness in developed and developing countries. Latest estimates from the National Eye Institute showed that 1100–1500 infants (~5% infants ≤1.25 kg at birth, <31 weeks of gestation) develop severe ROP that requires treatment. As these infants grow up, they have a higher incidence of astigmatism, high myopia, and retinal detachment and should be followed routinely afterwards. Unfortunately, recent estimates from the National Eye Institute showed that 400–600 infants (~2% infants weighing ≤1.25 kg at birth, 40% severe ROP infants) become legally blind from ROP despite treatment, translating to losing 30,000 life years of vision. The resulting long-term disability, and severely affected quality of life, in ROP patients poses an intense burden on the pediatrics, adolescent and adult healthcare system worldwide. As the population ages, high societal cost over many years is anticipated—making ROP a major public health issue and justifying a need for adequate management.

Current proven treatment for ROP is limited. Invasive retinal ablation is the most commonly used treatment, but it is inherently destructive to the retina. The lack of pharmacological treatment for ROP creates a great need for effective and safe therapies in these developing infants.

Neuroprotection in ROP is one way that may protect not only the retinal neurons, but also the vasculature. In the past years, many therapeutic targets have been developed, and they are targeted to vascular protection in ROP. In this review, we discussed the pathology and animal models of ROP, as well as the recent vascular protective and potential neuroprotective targets in ROP.

## 2. Vasculature in the Retina

### 2.1. Normal Development of the Retinal Vasculature

The survival and appropriate functioning of retinal cells depend on sufficient oxygen supply. Lack of oxygen may lead to vision loss. In the retina, normal visual process demands high energy, most of which are derived from oxidative metabolism coupled to adenosine triphosphate (ATP) synthesis. In the central nervous system, the brain receives 15% of the cardiac output and consumes around 20% of the total body oxygen, although it only represents 2% of the bodyweight [2]. Meanwhile, the consumption of oxygen in each gram of tissue in the retina has been described higher than that of the brain [3], making retina one of the highest oxygen-consuming tissues in the body [4]. Therefore, a well-organized vascular system providing adequate blood supply is important for the retina to maintain its normal function.

The blood supply of the retina is provided by two vascular systems: The retinal vessels for the inner two-thirds of the retina, and the choroidal system for outer one-third of the retina [5]. During development, the inner retinal vascular system undergoes significant changes and reorganizations. At the very beginning, tissues in the inner part of the eye are nourished by the hyaloid vasculature, which is a vitreous arterial network. In this hyaloid vascular system, blood enters through the central hyaloid artery initiating from the optic nerve, runs in hyaloid vessels and then exits through an annular collection vessel in the anterior part of the eye. The hyaloid vasculature is gradually replaced by the retinal vasculature as the development paces on [5,6]. The regression of hyaloid vasculature in humans occurs around mid-gestation, and in mouse occurs around birth [7]. At almost the same time as hyaloid vascular system regresses, retinal vasculature begins to emerge from the optic nerve head. This newly formed vascular plexus expands in the nerve fiber layer across the inner surface of the retina. Unlike the hyaloid vasculature, this plexus has both arteries and veins which go in and out of the optic nerve. Later, the primary vascular plexus gives rise to two other networks. Three of these vascular plexuses are parallel and inter-connected, which locates in the nerve fiber layer, inner plexiform layer and outer plexiform layer, respectively. In human beings, the normal retinal vascular development begins at around 16 weeks of gestation and accomplishes around birth. In mouse retina, the primary plexus reaches the periphery of the retina on about postnatal (P) day 8, and the three retinal vascular plexuses establish on about P21. The direction of vascular formation is from the center to the periphery; thus, vessels at the growing edge are less mature than those in the central areas. It is, therefore, possible to observe different stages of vascular differentiation in a peripheral to the central gradient.

### 2.2. Pathogenesis of Retinopathy of Prematurity

**Figure 1:**
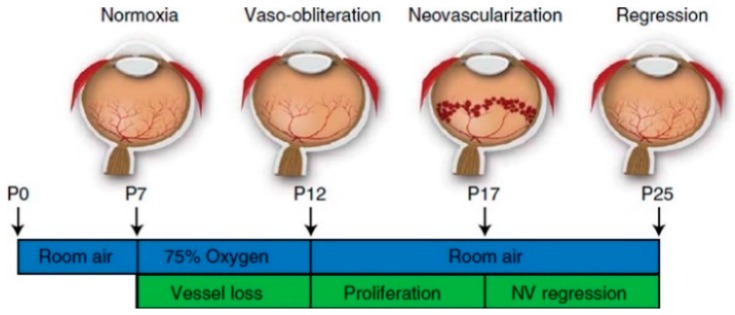
Schematic diagram of mouse oxygen-induced retinopathy (OIR) model [15]. The nursing mother and their pups were exposed to a 75% hyperoxia environment, which simulates the supplementary oxygen therapy in human. It results in vessel loss and presents features similar to those in phase 1 of human retinopathy of prematurity (ROP) development. Pups return to room air after P12. Hypoxia-induced neovascularization starts from P14 and maximizes at P17. Neovascular formation after hyperoxia in the mouse OIR model mimics phase 2 of human ROP development.

A major cause of blindness in children is ROP, a condition commonly found in preterm babies that are related to abnormal development of retinal blood vessels. Clinically, preterm infants are placed in high supplemental oxygen to facilitate breathing due to their immature cardiopulmonary system. The exposure to high oxygen (hyperoxia) during the first ischemic phase inhibits the production and secretion of pro-angiogenic factors, e.g., vascular endothelial growth factor (VEGF) and insulin-like growth factor-1 (IGF-1), while stimulating the formation of reactive oxygen species (ROS) [8,9]. As a result, apoptosis of vascular endothelial cells causes cessation of normal vessel growth and pruning of the existing immature vasculature, which, in turn, leads to retinal avascularity. The retinal response during this phase may also be mediated by hyperoxia-induced free radicals, although their roles remain unclear. Later, when the prematurely born infant acquires adequate cardiopulmonary functions, oxygen supplementation is discontinued, and the infant is returned to stay in normal room air. Now, relative retinal hypoxia occurs due to high oxygen demand from the maturing neural components. During this second vaso-proliferative phase, a compensatory release of pro-angiogenic factors is triggered. The expressions of VEGF, IGF-1 and erythropoietin (Epo) are increased, while hypoxia also facilitates the accumulation of hypoxia-inducible factor (HIF) [9]. There are now upregulated vessel growth and neovascularization (Table 1). These fragile neovascular tufts will, in turn, lead to intravitreal hemorrhages, retinal detachment and subsequent vision loss. Although poorly studied, the fluctuations in oxygen tension also predispose retinal neurons to degeneration (Figure 1). In fact, retinal dysfunction has been reported in infants and children with a history of ROP.

The etiology of ROP appears to be multifactorial. The severity of ROP is inversely proportional to gestational age, which is the greatest risk factor. A multi-centered national study, Supplemental Therapeutic Oxygen for Prethreshold Retinopathy of Prematurity (STOP-ROP) found that oxygen (96-99% saturation) does not cause additional progression of pre-threshold ROP [10]. However, there was absolutely no data to suggest that high oxygen level is safe for the early immature eye in the preterm infants that have not yet established ROP. On the other hand, it has been shown that the progression of ROP is closely related to the supplementary oxygen therapy that provided for the preterm babies after birth [11,12,13]. The lower oxygen concentration in the therapy has a beneficial effect on ROP development and can lower the severity of ROP [14]. However, if the preterm infant requires supplemental oxygen due to cardio-pulmonary reasons, withholding oxygen for fear of causing ROP is not recommended. Therefore, an understanding of tissue hyperoxia/hypoxia swing, as well as free radical and pro-angiogenic factor productions is integral to the prevention and treatment for ROP.

#### 2.2.1. Vascular Endothelial Growth Factor (VEGF)

VEGF plays for a key role in vascular development and angiogenesis [54,55]. There are three predominant VEGF isoforms in human (VEGF121, VEGF165 and VEGF189). There are two receptors common for all isoforms, which include fms-related tyrosine kinase 1 (FLT-1, also called VEGFR-1), and kinase insert domain-containing receptors (KDR, also known as FLK-1 or VEGFR-2). Meanwhile, heparin sulphate proteoglyvans (HSPGs), neurophilin 1 (NRP-1) and neurophilin 2 (NRP-2) are the receptors that only recognize the VEGF165 isoform [56]. Under hypoxia, the expression of *flt-1* gene is upregulated that leads to a higher binding efficiency between HIF-1α and VEGF promotor [57,58]. Unlike FLT-1, KDR is not induced by hypoxia, but it is important for normal early vascular development [59,60,61,62]. The main function of KDR is cell proliferation, differentiation, migration and maturation. There are several known VEGF sources in the human retina, including retinal pigmented epithelial cells, endothelial cells, astrocytes, Müller cells and other ocular tissues [63]. The retinal angiogenic changes under hypoxia are stimulated by the astrocytes release VEGF for vascular development toward the periphery [64], while the Müller cells release VEGF for vessel growth in the deep vascular plexus [65,66]. VEGF is one of the most important elements for ROP progression when it is under hypoxia condition.

#### 2.2.2. Insulin-Like Growth Factor-1 (IGF-1)

IGF-1 is a growth factor that is received maternally through the amniotic fluid and placenta during fetal development. IGF-1 plays a critical role in both normal retinal development and pathological ROP progression [67]. Despite a normal VEGF expression, IGF-deficient mice displayed an abnormal vasculature and slower vascular growth rate, indicating that IGF-1 is a key growth factor for vascular development in the early stage [67]. In the first stage of ROP, hyperoxia suppresses the expression of VEGF and IGF-1, whose levels control the survival of endothelial cells by the downstream Akt signaling [67,68]. Although a normal concentration of VEGF and IGF-1 supports the survival of endothelial cells, IGF-1 promotes apoptosis under hyperoxia in the first stage of ROP. In turn, this reduces vessel growth and give rises to an avascular retina.

Under hypoxia, the concentration of IGF-1 is gradually increased. IGF-1 can regulate the level of VEGF through different signaling pathways (such as p44/42 NARK pathway and P13K/Akt pathway) and results in neovascularization. Under various oxygen levels, IGF-1 has a different expression level and in combination with VEGF and HIF-1 can stimulate abnormal neovascular growth. A significantly high IGF-1 level inhibits the apoptosis of endothelial cells and promotes neovascularization by accumulating VEGF in the vitreous [68], resulting in ROP.

#### 2.2.3. Erythropoietin (Epo)

Epo is secreted from the fetal liver and adult kidney. This glycoprotein binds to the homodimer Epo receptors (EpoR) for inducing erythropoiesis, or binds to the heterodimer receptor, which consists of EpoR and common β receptor (Cβ-R), for another function. Epo has a beneficial effect on the experimental models of stroke and light-induced retinal degeneration [69]. Meanwhile, it also works as a mitogenic factor for endothelial cells of brain capillaries. Epo production can be stimulated by hypoxia, thus, induces angiogenesis. VEGF and Epo have a direct relationship, and both are stimulated by HIF-1α. Under hypoxia, not only the expression of VEGF is upregulated, but also Epo [70]. VEGF and Epo have similar functions in vascular proliferation. In the Epo-deficiency animal model, the vascular loss and neovascularization formation were both reduced in the retina that, in turn, decreased the disease damage in oxygen-induced retinopathy (OIR) [71]. In addition, the level of Epo is directly correlated with the level of VEGF and plays a similar role in endothelial cell proliferation in the second stage of ROP.

#### 2.2.4. Hypoxia-Inducible Factor-1 (HIF-1)

HIF-1 contains α and β subunits. This heterodimer plays a critical part in body regulation under hypoxia [72]. HIF-1α is a short-lived nuclear protein. Under normal condition, HIF-1α is hydroxylated and degraded by prolyl hydroxylase (PHD). However, in a low oxygen environment, HIF-1α is not hydroxylated, and PHD is no longer efficient, thereby stimulating the accumulation of HIF-1α inside the nucleus. The combination of α and β subunits are then facilitated, which, in turn, binds to the hypoxia response element (HRE) in the VEGF promotor region. Sufficient accumulation of HIF-1α under hypoxia induced VEGF production [56,72]. Therefore, there is a direct relationship between hypoxia, HIF-1α and VEGF expression.

#### 2.2.5. Nitric Oxide (NO)

NO is the core element for the signaling process that is synthesized by NOS (nitric oxide synthetase). Two isoforms of NOS work in different roles by different manners, which include the constitutive (cNOS) and inducible (iNOS). The cNOS, such as endothelial (eNOS) and neuronal NOS (nNOS), synthesizes NO when the secretion of calcium is upregulated. Unlike cNOS, iNOS is a calcium-independent enzyme and is only found in specific tissues [56]. The regulation of NO depends on the stimulation or suppression of the specific NOS.

The change in oxygen concentration affects the expression of eNOS, as well as NO concentration. NO level is lowered when the expression of eNOS is reduced during hyperoxia. It promotes the inhibition of proliferation and formation of avascular retina [73,74]. Under hypoxia, eNOS expression is stimulated, which, in turn, increases the NO concentration and induces angiogenesis [75]. The NO production during the hyperoxia-hypoxia induction is correlated with the severity of ROP.

#### 2.2.6. Adenosine

Adenosine is an endogenous nucleotide and acts as a neuromodulator. In the retina, it is generated during AMP hydrolysis by 5′ nucleotidase (5′ N) in the Müller cells. Adenosine release is mainly caused by stress, tissue activity, or hypoxia. The level of adenosine is also changed under different oxygen level as the activity of 5′ N is suppressed under hyperoxia and is induced under hypoxia. Adenosine regulates different cell functions by activating different adenosine receptors which consist of adenosine 1 receptor (A_1_R), adenosine 2A receptor (A_2A_R), adenosine 2B receptor (A_2B_R) and adenosine 3 receptor (A_3_R) [76]. A_2A_R is closely related with neurodegeneration [77] and demonstrated a positive effect after blockage of A_2A_R in neurodegenerative models, such as Parkinson disease (PD) [78,79], Alzheimer disease (AD) [80] and ischemia [81]. This receptor interferes with microglial-mediated inflammation that, in turn, induce neuronal damage by stimulating NO production from the microglia-derived mediators [82]. Therefore, more adenosines are released and subsequently stimulates neuronal death and vascular endothelial cells proliferation [83,84,85] by microglial-mediated inflammatory responses in the second stage of ROP. Recently, several studies demonstrated the association between early microglial activation and retinal ganglion cell death in a glaucoma model and indicated that these two events were initiated simultaneously and contributed to the progression of neurodegenerative diseases [79,82,86,87]. Therefore, there is a direct relationship between A_2A_R stimulation, microglial activation and apoptosis in retinal ganglion cells in the onset of neurodegenerative diseases.

#### 2.2.7. β-Adrenergic Receptor (β-AR)

Adrenergic receptors (AR) play a role in an internal regulatory manner that is mediated by adrenaline and noradrenalin. Two types of adrenergic receptors, α-and β-AR, tend to have a systemic response after binding with its agonists. α-AR promotes the action of muscle contraction, and insulin suppression, as well as induces platelet aggregation. Unlike, α-AR, β-AR has totally opposite effects, such as muscle relaxation and induction of the secretion of insulin, VEGF and renin [72]. The concentration of β-AR is higher during aging and hypoxia [88]. Cell proliferation is also induced by the stimulation of β-AR under hypoxia [89]. Recent studies indicated that β-AR was overexpressed in the vascular endothelial cells [90] and regulated the neovascular formation. This evidence suggests that β-AR has a role in abnormal neovascular formation and responses to ischemia and hypoxic condition.

## 3. Animal Models for ROP—Oxygen-Induced Retinopathy (OIR)

It is an ethical problem for drug testing in human preterm infants; therefore, an animal model is desirable for studying the mechanism and possible therapy for ROP. OIR is an in vivo method for mimicking human ROP. The animals that are used in OIR are full-term neonates with an immature retinal development at birth so that further retinal development under various experimental conditions can be monitored. Although animal models are one way to investigate ROP, they cannot fully simulate the situation in human. Human preterm babies usually have complications after birth, such as bronchopulmonary dysplasia, sepsis and necrotizing enterocolitis, which are seldom observed in the full-term newborn animals. Among the various animal models for ROP, the duration and oxygen level provided for inducing avascular zone and neovascularization are different in different animals. Among these, mouse and rat OIR models are the most common animal models to study ROP.

A mouse OIR model was generated in the 1990s by Smith and colleagues for studying the pathogenesis of ROP [91,92]. This OIR model is one of the commonest models in ROP study. Mouse neonates and their lactating mother are exposed to high oxygen (75%) environment from P7 to 12, and then returned to room air to induce OIR. After hyperoxia-hypoxia induction, a large central avascular zone is seen on P12, and a peak neovascularization is observed on P17 (Figure 1). There are a number of advantages that the mouse OIR model can offer. Firstly, it is convenient to use a stable oxygen level for five days. Secondly, it is easy to obtain transgenic mice with OIR for investigating the mechanism of ROP. However, mouse OIR model does not completely represent all the pathologies seen in human ROP as mentioned above. Besides the various complications seen in human preterm babies, newborn mice have higher arterial oxygen after OIR when compared with human infants. In addition, the avascular zone is induced in the central retina in mice, but a peripheral avascular retina is actually observed in human [34]. Although there are some limitations in the mouse OIR model, the mouse is an animal that is easy to handle for further studies the pathogenesis of ROP.

Another OIR model that is commonly used is the rat model. Rat OIR model was designed by Penn in 1993 [93]. Unlike the mouse OIR model, a hyperoxia-hypoxia cycle by fluctuating oxygen level is utilized for inducing avascularization and neovascularization in rat. The oxygen-controlled environment is changed from 50% to 10% oxygen for 14 days after birth (from P0 to P14) and is changed every 24 h. One advantage of this rat model is its closeness to human ROP. Both rat and human have similar arterial oxygen. In addition, the appearance of OIR induced retinal vascular development is similar to type 1 severe ROP. As in human, rats have a peripheral avascular retina after OIR. However, the availability and usage of transgenic rats are limited. The molecular mechanism or pharmacologic pathway is also more difficult to analyze using the rat OIR model.

## 4. Vascular Protection in the ROP

Over the past 80 years, many ROP studies have been concentrated on vascular changes. The measurements of avascular and neovascular areas are the comparative method for identifying the protective roles of agents. Many therapeutic studies in ROP would conclude a vascular protective effect when a reduction of avascularization and neovascularization is observed, but there is very limited data on neuronal responses after treatment.

**Figure 2:**
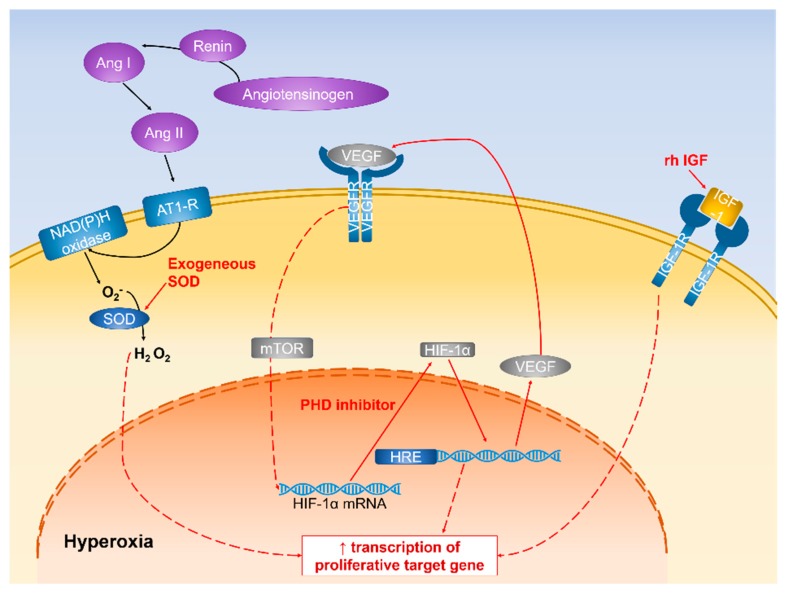
Schematic diagram representing the mechanism of vascular changes in hyperoxic condition. Pathways that confer vascular protection are highlighted in red.

**Figure 3:**
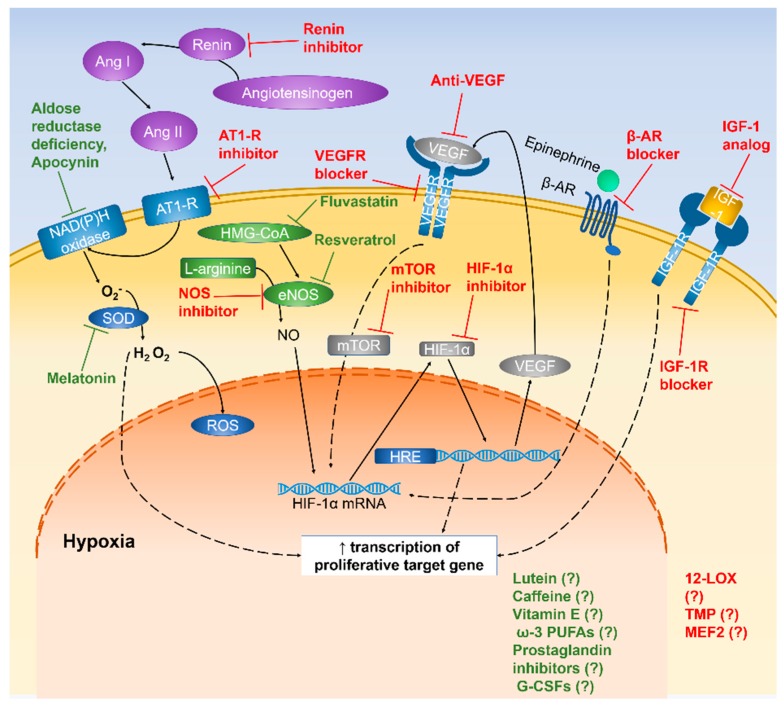
Schematic diagram representing the mechanism of vascular changes in hypoxic condition. Pathways that confer vascular protection are highlighted in red and neuroprotective pathways are highlighted in green.

Many components are responsible for the internal regulation of retinal vessel development under hyperoxia or hypoxia. Hypoxia-induced oxidative stress is the common product of imbalance between prooxidants and antioxidants. Some oxygen-sensitive agents, such as VEGF, IGF, and HIF-1α, have pathological roles in ROP progression; yet, they might have a beneficial effect on vascular changes if they are inhibited or induced during ROP development (Figure 2 and Figure 3).

### 4.1. Growth Factors

#### 4.1.1. Anti-VEGF

VEGF level plays a pathological role in both phase 1 and 2 of ROP. The therapeutic role of VEGF was firstly described in 1995 [53]. VEGF was intraocularly injected during hyperoxia, and this prevented the apoptosis of vascular endothelial cells, resulting in the reduction of avascular area in the rat OIR model. As vessel regression was indicated when there was increased VEGF concentration during hyperoxia, anti-VEGF was then suggested as a potential agent for vascular protection. In anti-VEGF therapy, antibodies attach to VEGF, thus, reducing its own binding to its receptors and the following angiogenic effects. Currently, bevacizumab (Avastin), ranibizumab (Lucentis) and aflibercept (Eylea) are three types of anti-VEGF agents for ROP patients [54,55,59,60,61]. They have different designs for prolonging the efficiency and eliminating inflammatory responses. Recent animal studies and clinical trials for these three drugs displayed a beneficial effect on intravitreal injection of anti-VEGF in reducing neovascular area and continuing vessels growth toward the peripheral retina after treatment [54,61,63]. However, there might be general effects despite a drop in systemic VEGF level after injection [55,56]. As the blood-retinal barrier (BRB) is broken down during hypoxia, the injected anti-VEGF agents can enter the vascular system, and in turn, bind to the circulating VEGF. The sudden reduction in VEGF level may indicate a long-term effect of neurodevelopment [59,60]. Although there may be an unknown lifelong effect resulted from an anti-VEGF injection, it is the only efficient treatment for eliminating neovascularization growth in the severe ROP infants now.

Besides these three well-established drugs, other VEGF-related inhibitory agents have been proposed. Short hairpin RNA linked VEGF (VEGFA shRNA), anti-KDR antibody, SRPIN340 and rapamycin were investigated in the suppression of neovascular development. Gene silencing of VEGF could significantly reduce VEGF expression in rat OIR model using VEGFA shRNA injection and also inhibit neovascularization in both short-term and long-term studies [64]. Blockage of the VEGF receptor is another direct way to suppress VEGF activities. KDR is one of the VEGF receptors in human. Anti-KDR did not affect normal vascular development, but suppressed neovascularization in the OIR-treated group [65]. Moreover, SRPIN340 and rapamycin were proposed for indirectly reducing VEGF expression. SRPIN340 is the inhibitor of serine arginine protein kinase 1 (SRPK1), which plays a role of phosphorylation of SRSF1 and VEGF165 splicing. The phosphorylated serine-rich splicing factor-1 (SRSF1) translocates to the nucleus and induces alternative splicing, resulting in upregulation of VEGF. The injection of SRPIN340 significantly decreased VEGF concentration in OIR-treated rats [66]. Rapamycin has an anti-angiogenic effect by inhibiting the mammalian target of rapamycin (mTOR), which is the VEGF induced pathway. The neovascular area was reduced after rapamycin-treated in OIR mice [67]. Through either a direct or an indirect way of VEGF inhibition, the anti-VEGF agents could successfully eliminate the activity of hypoxia-induced upregulation of VEGF.

#### 4.1.2. IGF-Binding Protein (IGFBP)

Based on the understanding of IGF expression in ROP progression, the therapeutic targets are either increasing IGF level in phase 1 or reducing IGF concentration in phase 2. IGF concentration was enhanced by injection of recombinant human IGF-1 (rhIGF-1) before hyperoxia [68]. The mouse pups that received rhIGF-1 were heavier and had a higher score in maturation assessments, including the appearance of black skin color and eye opening. They also had lower neovascularization comparing with placebo. For reducing IGF level in the hypoxia phase, IGFBP3 and Jb3 were investigated. In the normal situation, free IGF-1 tends to bind with the IGF-1 receptor on the cell surface and induce angiogenesis. If there is more IGFBP3 in the extracellular fluid, IGFBP3 will bind with IGF-1. The downstream process of IGF-1 will then stop, and this will trigger cell apoptosis. Therefore, a reduction of neuronal cell apoptosis was indicated in the IGFBP3-injected OIR mice [69], and a larger retinal avascular zone was observed in the IGFBP3-deficient mice [70]. Jb3 is the inhibitor the of IGF-1 receptor. Similar to the action of IGFBP3, Jb3 bind to IGF-1 receptor and block its binding site of IGF-1. Jb3 injection in mouse model affected the vascularization and resulted in fewer neovascular tufts after hyperoxia-hypoxia induction [71]. Either induced or suppressed IGF level in ROP phase 1 or 2 have a beneficial effect on vascular development and maturation.

### 4.2. Transcription Factors

#### 4.2.1. Regulation of HIF-1α Expression

The increased or reduced HIF-1α concentration is one way to attenuate the condition of vessel loss in phase 1 or neovascularization in phase 2, respectively. Prolyl hydroxylase (PHD) plays a role in the degradation of HIF-1α and suppression of its activity. The PHD inhibitor, dimethyloxalylglycine (DMOG), and PHD2-deficient mice demonstrated an accumulation of HIF-1α and prevention of vessel loss and vessel tufts in the early and late stage of OIR [72,73]. The suppression of HIF-1α was also investigated using RTP801-deficient mice. RTP801 is a novel responsive gene of HIF-1. It is upregulated under hypoxia and induced the transcription of HIF-1. After OIR, RTP801-deficient mice displayed an attenuated neovascular tuft and decreased apoptosis [74]. Another method to reduce HIF effects is the direct inhibition of HIF by topotecan and doxorubicin that suppress the accumulation of HIF-1α protein [94] and block HIF binding with its hypoxia-response element [95], respectively. Both inhibitors significantly decreased neovascular tufts in the mouse OIR model, and retinal function was further protected by topotecan treatment [96]. The use of upregulation or downregulation in HIF-1α has a significant effect on suppression of ROP development.

#### 4.2.2. Inhibitory Effect of NOS Expression

Inhibition of NOS can suppress cell proliferation and neovascular formation. For eliminating the NOS expression on phase 2 of ROP, the pretreatment of NOS inhibitors was investigated. *N*-nitro-l-arginine (l-NA), *N* ^G^-nitro-l-arginine (l-NNA), aminoguanidine (AG) were injected into the animal during hyperoxia [75,76,77]. l-NA and l-NNA are the general NOS inhibitors, and AG is the specific iNOS inhibitor. Studies demonstrated a reduction of the avascular zone and neovascular tufts in the NOS and iNOS inhibitor groups. The elimination of NOS expression plays a protective role in retinal damage.

#### 4.2.3. Blockage of β-ARs

Vascular changes in ROP progression partially depend on β-AR concentration in the 2 phases of ROP. Propranolol is a nonselective β-AR antagonist. The vascular protective effect in propranolol is quite controversial. Subcutaneous injection (0.02 to 20 mg/kg/dose) or topical administration (0.5 to 20 mg/kg/dose) of propranolol after hyperoxia resulted in an inhibition of neovasculature and VEGF expression [78,79]. Similarly, another investigation administrated propranolol (2 to 60 mg/kg/dose) after hyperoxia by oral, intraperitoneal, or subcutaneous injections [97]. However, these three propranolol-treated groups showed no significant difference in avascular and neovascular areas, as well as VEGF level. Moreover, a recent investigation in the use of propranolol in the mouse OIR model demonstrated an exacerbation of OIR where higher pericyte apoptosis and vascular permeability were observed [98]. On the other hand, the blockage of three β-ARs subtypes (β_1_-, β_2_- and β_3_ -ARs) using their respective specific inhibitor, atenolol, ICI 118,551 and SR59230A showed different results [80]. The β_2_-AR blocker has a remarkable protection in vascular changes and a better retinal function after OIR. Therefore, the impact of using β-ARs blockers is still unclear, and more studies are required in investigating its protective properties after OIR.

### 4.3. Anti-Angiogenesis

Uncontrolled angiogenesis is a factor of neovascularization in phase 2 ROP. Anti-angiogenic agents provide a way to disrupt the vasculature formation pathway, thereby inhibiting abnormal neovasculature formation in ROP.

#### 4.3.1. Steroid Agents

Dexamethasone and anecortave acetate were identified as anti-angiogenic agents, but it may not be a suitable drug for ROP therapy. Dexamethasone and anecortave acetate were administrated in the mouse and rat OIR studies and showed a beneficial effect in neovascular suppression [81,82]. However, a recent clinical investigation mentioned that postnatal steroid use has a higher risk of severe ROP and fungal sepsis development [99]. Despite their roles as anti-angiogenic agents, dexamethasone and anecortave acetate are in fact independent risk factors of ROP severity.

#### 4.3.2. Other Angiogenic Inhibitors

Some other anti-angiogenic agents have been discussed and exhibited the suppression of neovascular formation in rat or mouse OIR model. They have a common feature of suppression of VEGF, IGF-1, HIF-1α and other angiogenic factors. For example, deguelin, YC-1 and β- lapachone are the components that indirectly regulate the HIF-1α expression [83,84,85]. Glial water channel aquaporin-4 (AQP4), linagliptin, furin and desmogleins are able to inhibit VEGF expression or its activity from reducing the effect of OIR [100,101,102,103]. *N*-terminal fragment of human prolactin (16K HPRL) and 12-lipoxygenase (12-LOX) mediate endothelial cell proliferation [86,87]. Tetramethylpyrazine (TMP), plasminogen kringle 5 (K5), myocyte enhancer factor 2 C (MEF2C), anti-secretogranin III, mini-peptide ribosomal protein L41 (RPL41) and valproic acid have a protective and preventive effect in vascular diseases [88,89,90,104,105,106].

However, less information and investigations in ROP support was found for these anti-angiogenic components. More studies will be needed for further evaluating their protective effect on ROP.

## 5. Neuroprotective Agents in ROP

ROP can also be considered one of the neurodegeneration diseases, and it happens when the infants received supplementary oxygen therapy. The presence of hyperoxia-hypoxia induction is harmful to the retinal neurons due to the presence of oxidative stress. Moreover, the hypoxic damage causes the production of free radicals, inadequate blood supply and other inflammatory actions, as well as the apoptotic effect in neuronal cells [107]. Many investigations proposed the use of anti-oxidative and anti-inflammatory agents for possible prevention of neuronal apoptosis, and therefore, as treatments for ROP (Figure 1).

Moreover, recent evidence showed that neurodegeneration might occur before the vascular damage in diabetic retinopathy (DR) [108]. Similar to DR, ROP is also a kind of ischemic retinopathies. Although there is no any direct evidence showed that the progression of neovascularization in DR and ROP are the same, many researchers have studied retinal neovascularization in the proliferative DR using OIR model [8,15,109,110,111,112,113,114]. They may have a similar aspect of vascular changes in DR and ROP, but very limited studies have examined the association between neurodegeneration and vascular changes in OIR or ROP. Therefore, we hypothesize that neuroprotective agents may potentially be a promising therapeutic strategy for ROP.

### 5.1. Antioxidants

Antioxidants are the agents that have the ability to reduce oxidative stress. They can be divided into nutritional and systemic antioxidants. The antioxidants that can be obtained from the dietary sources are classified as nutritional antioxidants. Some food components have the properties of blocking the oxidative pathway or suppressing free radical production. Therefore, the consumption of these foods may prevent oxidative stress, and thus, neuronal damage. Another group of antioxidants is the agents that are present in the body and play a role in the inhibition of oxidative stress, but are suppressed in hypoxia naturally. Both nutritional and systemic antioxidant are critical components for suppressing oxidative stress and enhancing the ability of neuroprotection.

#### 5.1.1. Nutritional Antioxidants

##### Lutein

Lutein has an anti-oxidative effect on the ischemic and hypoxic models [115,116,117,118,119]. Lutein is a class of xanthophyll carotenoid and is contained in the dark vegetables and fruits, such as broccoli, kale and kiwi fruit. It cannot be generated in the body, and must, therefore, be ingested. Lutein is very safe. It is approved by the Food and Drug Administration (FDA) and considered as GRAS (generally recognized as safe). In the eye, it is present in the macula and lens, and acts as a photoprotective agent in screening out the harmful blue light [120,121]. Besides, lutein is an antioxidant and potential preventing agents for cataract and age-related macular degeneration (AMD) [120]. Photo-oxidative damage leads to protein oxidation, whose products can precipitate in the lens and results in cataract. Although there is a smaller amount of lutein concentrated in the lens (compared to the macula), it is possible to block the high energy blue light and prevent cataract. Similarly, in AMD, the presence of lutein suppresses the extent of photo-oxidation, and thus, reduces the formation of neovascularization [120,122,123]. In addition, lutein is shown to have anti-inflammatory properties in attenuating the activity of NF-κB [118,124]. Lutein provides neuroprotective effects in both in vitro and in vivo studies. Higher cell viability and suppressed inflammatory responses were shown in the lutein-treated retinal ganglion cells (RGC-5) and rat Müller cells (rMC-1) after chemical-induced hypoxic damage [124,125]. It also prevents neuronal damage in mouse ischemia/reperfusion (I/R) injury model [117,118,124], rat retinal detachment (RD) model [126] and rat *N*-methyl-d-aspartic acid (NMDA) retinal damage model [127]. When administrated 1 h before and 1 h after reperfusion in the I/R injury model, lutein treatment yielded a better neurological scoring, less brain damage, such as smaller infarct area and infarct volume, suppression of oxidative stress by inhibiting NFκB signaling pathway, and inhibition of apoptotic pathway by increasing phosphorylation of Akt and Bcl-2 expression. Lutein’s retinal protective effect exhibited in the RD and NMDA models showed fewer TUNEL-positive cells, reduced GFAP immunoreactivity and inhibited apoptotic effect in RGC. In studies on the effect of lutein in the mouse OIR model, lutein was daily injected to mice during hypoxia. Interestingly, lutein treatment did not only reduce avascularization and vascular leakage, but also protected astrocytes and promoted the formation of endothelial tip cells [128]. Lutein may, therefore, be a potentially beneficial agent for maintaining vasculature and neuronal cells in human ROP.

##### Caffeine

Caffeine is commonly found in coffee, cola and tea. It serves as a stimulant in the central nervous system. Its anti-oxidative characteristic was first described in 1991 [129]. Earlier investigations mentioned the protective effect in the inhibition of oxidative DNA damage, attenuation of oxidative stress in rat liver and anti-inflammation by regulating TNF-alpha production [130,131,132,133]. In addition, caffeine (when cotreated with adenosine A2A receptor antagonists) displayed a neuroprotective effect by acting against β-amyloid-induced neurotoxicity in an in vitro AD study [134]. Recently, the neuroprotective effect of caffeine was indicated using a rat hyperoxia injury model where caffeine was injected intraperitoneally before exposure to 80% oxygen environment [135]. Fewer TUNEL-positive neuronal cells were observed in the caffeine-treated immature brain samples. Caffeine was then investigated in the mouse OIR model. The water-soluble caffeine was administered by addition to the drinking water. This treatment did not affect normal vascular development, but it could eliminate avascular area and neovascular tufts. The concentration of VEGF was significantly lower than that in the untreated group with a lower apoptotic response in neuronal cells after treatment [136]. Caffeine, therefore, has a beneficial effect on the prevention of neuronal damage in OIR. However, the adverse and long-term effect of caffeine in premature infants are not discussed; this can potentially cause caffeine addiction after treatment.

##### Omega-3 Long-Chain Polyunsaturated Fatty Acids (ω-3 PUFAs)

Long-chain eicosapentaenoic (EPA) and docosahexaenoic (DHA) fatty acids are the ω-3 PUFAs that have a beneficial effect of oxidative stress prevention. Omega-3 is enriched in fish oil. The protection of neuronal cells using ω-3 PUFAs has been shown in many neurodegeneration diseases, such as AD and PD. In the epidemiological studies conducted in France and Chicago, participants who have a higher consumption of fish or DHA have a lower risk of dementia or AD [137,138,139]. To further understand the effect of PUFAs, transgenic mouse model, Tg2576 mouse, was generated for investigation of neurodegeneration in AD. The DHA-treated mice have a lower apoptotic response and fewer behavioral deficits in the swimming behavioral test [140]. After that, the protective effect of ω-3 PUFAs and DHA was investigated in the in vitro RGC-5 cells or in vivo mouse OIR model. RGC-5 cells were protected by DHA under H_2_O_2_-induced oxidative stress by inhibition of apoptosis [141]. In the animal studies, the diet was mixed with ω-3 PUFAs and fed to the lactating mother during hyperoxia. Neovascularization was significantly inhibited after ω-3 PUFAs treatment [142,143,144]. Although the neuroprotective effect of EPA and DHA was showed in the in vitro RGC cells, it is essential that more studies are required to support its neuroprotective effect in the in vivo OIR models.

##### Resveratrol

Resveratrol is a phytoalexin that can be absorbed from the dietary sources, such as grapes, berries, and peanuts. It is a natural antioxidant by reducing the generation of ROS and maintaining the concentration of intracellular antioxidants [145,146,147]. Other than being an antioxidant, it also contains anti-inflammatory properties [148,149]. It provided a neuroprotective role in animal models for AD, PD, and stroke [146,150,151,152,153,154,155]. Decreased apoptosis of neuronal cells and prevention of motor impairment were observed in the animal models for these neurodegenerative diseases. Resveratrol also showed a beneficial effect in in vitro and in vivo rat OIR studies. Resveratrol was provided during hyperoxia in the in vitro OIR primary culture study that resulted in I attenuated eNOS and nNOS levels after treatment [156]. It was then further investigated in a rat OIR model. Resveratrol injection was performed either during hyperoxia or after hyperoxia intragastrically. The concentration of eNOS and nNOS, as well as Bcl-2 expression, were suppressed in the resveratrol-treated groups [156,157]. Resveratrol might have an anti-angiogenic effect in the OIR model by inhibiting the several angiogenic factors. Despite resveratrol’s potential neuroprotective properties in AD, PD and stroke models, there is a lack of information for its beneficial effects in OIR models.

##### Vitamin E

Vitamin E is a well-known fat-soluble antioxidant and is commonly found in vegetable oil, meat and eggs. It can serve as a supplement for preventing cancer and heart disease, as well as treating diabetes. The neuroprotection pathway of vitamin E was still unclear, but the suppression of neurodegeneration has been shown in several studies. Neuronal damage was reduced in the animal ischemic model [158], and the suppression of AD progression was observed in a clinical trial [159]. More importantly, protective effects were first observed in the OIR model in 1977. Vitamin E was administrated to the pups by intraperitoneal injection or fed to their mother during hyperoxia [160,161]. The retinal vasculature of the pups had a much smaller avascular area after hyperoxia induction. In a clinical trial, there was also a reduction of ROP incidence after vitamin E treatment, and no adverse effects were identified [162,163,164,165]. However, there is currently no data to support the neuroprotective role of vitamin E in OIR, despite its well-established effects in the central nervous system.

#### 5.1.2. Endogenous Antioxidants

##### Suppression of Aldose Reductase

Aldose reductase is the first enzyme in the polyol pathway which converts glucose to sorbitol. Upregulation of polyol pathway directly induces oxidative stress under hyperglycemic condition [112,166,167,168,169,170]. Inhibition of aldose reductase has a protective effect on diabetic and ischemic models by reducing oxidative stress and VEGF expression, as well as preventing BRB breakdown [171,172,173,174]. In OIR, inhibition of aldose reductase expression and its association with OIR has been investigated using aldose reductase-deficient mouse model or administration of an aldose reductase inhibitor, Fidarestat [175,176]. The absence of aldose reductase in the mice does not affect normal retinal development, including vasculature, function and morphology. Interestingly, there was attenuation of avascular and neovascular areas and prevention of vessel leakage in the ganglion cell layer and outer plexiform layer in the aldose reductase-deficient mice and Fidarestat-treated retinae after OIR. Lower expression of proinflammatory response protein p-I_K_B and cell proliferative signaling molecular, such as VEGF and p-Akt was found with a milder GFAP immunoreactivity and microglial activation. More importantly, the absence of aldose reductase preserved retinal function after OIR. This neuroprotective role of aldose reductase deficiency in the OIR model was reflected in its anti-oxidative and anti-inflammatory events, thereby attenuating glial cell responses and neuronal cell death.

##### Superoxide Dismutase (SOD)

SOD is a water-soluble endogenous enzyme with anti-oxidative effect. It is the first line of oxidation defense by reduction of superoxide radicals. Natural SOD has large molecular weight, a short life-span, and lower availability in circulation. For prolonged life-span and increased activity, SOD is usually modified before exhibiting its protective effects, such as nonpeptidyl Mn-based SOD (MnSOD), liposomal SOD, and copper-zinc SOD (CuZnSOD) [177,178,179,180]. Neuroprotection by SOD was shown in the mouse stroke model of transient occlusion of the middle cerebral artery (MCAO) [177,179,181]. Mice after MCAO have an improvement of neurobehavioral outcome and reduction of infarct volume after injection of SOD mimetics and MnSOD. Furthermore, there was a reduction of intracellular superoxide radical concentration, and a increase in neuronal cell viability in in vitro primary culture. The effect of SOD overexpression was evaluated in mouse OIR model by the transgene of SOD gene or intraperitoneal injection of liposomal SOD and CuZuSOD [178,180]. These investigations showed no adverse effect in normal retinal development, but reduced avascular and neovascular areas after hyperoxia-hypoxia induction. Therefore, the anti-oxidation property of SOD provides prevention of ROP progression, but its effects on the retinal neurons, and hence, neuroprotection properties remain unclear.

##### Statin

Statin is a lipid-lowering drug that can prevent cardiovascular diseases [182]. It suppresses HMG-CoA reductase, the enzyme for biosynthesis of cholesterol [183]. Its prevention of neuronal cell death has been shown in both cell culture and animal studies. However, statin has a dose-dependent neuroprotective or neurotoxic effect in different studies [184]. Therefore, consideration of statin concentration becomes a critical part of treatment. Simvastatin, lovastatin, atorvastatin and fluvastatin are the common statins that are used as drug treatment for hypercholesterolemia [185]. Simvastatin (50 mg/kg/day) treatment caused an upregulation of Bcl-2 in gene and protein level and promoted an anti-apoptotic effect in mice and guinea pig AD model [186,187]. Fluvastatin, when administrated to the mouse intraperitoneally (10 mg/kg/day), suppressed the formation of retinal neovascularization, expression of VEGF, HIF-1α, inflammatory mediator ICAM-1, and elimination of superoxide production [188]. These studies showed that statin plays an anti-oxidative, anti-inflammatory and anti-angiogenic role in the OIR models; however, there is no mention of any protective effects on the retinal neurons.

##### Melatonin

Melatonin is a neurohormone derived from the pineal gland and is widely used in clinical applications. It provides regulation of normal body rhythms as a sleep aid supplement. Melatonin also contributes to stroke and some neurological disorders, including AD and PD. Melatonin-treated rats showed significantly reduced inflammatory responses, BRB permeability, and formation of cerebral edema after experimental stroke [189,190,191,192]. It also has a protective effect in neurodegeneration diseases by acting as a free radical scavenger. The anti-oxidative activity of melatonin is shown in the reduction of free radical and upregulation of antioxidant enzyme, including SOD, glutathione peroxidase and glucose-6-phosphate dehydrogenase [193,194,195]. Melatonin also provides beneficial effects in hypoxia-induced rat model and mouse OIR model [196,197]. Melatonin could reduce neovascularization by suppressing VEGF and HIF-1α secretions. Besides its role as a regulatory hormone, melatonin also offers neuroprotection in stroke and neurodegeneration. Unfortunately, its neuroprotective effects in the OIR model have not been investigated.

##### Apocynin

Apocynin is a NAD(P)H oxidase inhibitor, which was first isolated from *Apocynum* species. There is a direct relationship between NAD(P)H oxidase, ROS production, oxidative stress and progression of neurodegenerative disorders. NAD(P)H oxidase is the major source of ROS production. The accumulation of ROS leads to oxidative stress and causes neuronal damage, resulting eventually in neurodegenerative diseases. By inhibiting NAD(P)H oxidase, apocynin suppresses oxidative stress and prevents neurodegeneration [198]. Apocynin is a powerful blocker for NAD(P)H oxidase. It is easily oxidized by peroxidases and forms dimer or trimer derivatives [198]. The neuroprotective function of apocynin was well described in stroke and PD studies by yielding a better neurological outcome and reduction of oxidative stress and inflammatory responses [199,200,201,202,203,204]. The administration of apocynin has been proposed for treating OIR mice [205,206]. When administered by intraperitoneal injection after hyperoxia, beneficial effects in vascular protection, including suppression of neovascular formation and VEGF concentration were observed. Apocynin exhibits a protective effect in the OIR model as an indirect suppressor for oxidative stress, but no information is available on its neuroprotective roles in OIR.

### 5.2. Anti-Inflammatory Agents

#### 5.2.1. Prostaglandin Inhibitors

Indomethacin and ibuprofen are nonsteroidal anti-inflammatory agents and prostaglandin inhibitors. Many studies using these prostaglandin inhibitors are carried out for treating patent ductus arteriosus (PDA). PDA is a common heart disease in preterm infants, and it increases the risk of pulmonary hemorrhage, necrotizing enterocolitis (NEC) and intraventricular hemorrhage [207]. These prostaglandin inhibitors inhibit cyclooxygenase (COX), which can induce inflammatory cells. Other than the anti-inflammatory effect, they also exhibited the neuroprotective effect in brain injury and PD diseases by suppressing neuronal apoptosis, lipid peroxidation and superoxide production [208,209,210,211,212]. Specifically, subcutaneous administration of indomethacin and ibuprofen provides vascular protection in OIR [213,214]. The size of the neovascular area was significantly inhibited in the drug-treated OIR mice. These prostaglandin inhibitors not only provide a neuroprotective effect on brain injury and PD, but also play a role in vascular protection in OIR. Unfortunately, whether they can exert neuroprotective effects in OIR remains unclear.

#### 5.2.2. Granulocyte Colony-Stimulating Factor (G-CSF)

G-CSF is a well-known hematopoietic glycoprotein. It stimulates proliferation, survival and maturation of cells [215]. Many studies indicated that G-CSF promotes anti-inflammatory, anti-apoptotic and neuroprotective effects [216,217,218,219,220]. The presence of G-CSF reduces inflammatory responses and neuronal cell damage in both in vitro and in vivo rodent ischemic models. These beneficial effects were also shown in the H_2_O_2_ induced human retinal endothelial cell culture and mouse OIR model [221]. OIR-treated mice, receiving an intravitreal injection of G-CSF after hyperoxia, displayed suppressed neovascularization. Although G-CSF could suppress neuronal damage in ischemia models, there is limited data on its neuroprotection in the OIR model.

### 5.3. Others

#### Inhibition of Renin-Angiotensin System (RAS)

RAS regulates angiogenesis. Renin and angiotensin II type-1 receptor (AT1-R) play potential roles to either induce or suppress cell proliferation path in RAS. Renin is the initiation enzyme of RAS. Aliskiren, a renin inhibitor to attenuate renin secretion, was injected to the OIR-treated mice after hyperoxia [222]. Reduced retinal neovascularization and suppressed VEGF mRNA and protein level were observed in the aliskiren-treated OIR mice. In addition, other investigations suggested that blockage of AT1-R is another possible therapy for anti-angiogenesis in ROP, as AT1-R can stimulate cell proliferation, fibrosis, and angiogenesis. Valsartan is an AT1-R inhibitor [223,224]. After valsartan treatment by intraperitoneal injection in the rat, it prevented the neovascular formation and had a neuroprotective effect. Treatment with valsartan yielded an extensive glial vascular network and enhanced astrocytes coverage in the retina after OIR, indicating that blockage of RAS may be a potential therapy for ROP.

## 6. Stem Cell Therapy in ROP

Stem cell therapy is now a popular approach to treat a disease. Stem cells (SCs) have the characteristics of differentiating into specialized cell types with unlimited renewing properties [225]. These properties provide great potentials for therapeutic uses in tissue repair and regeneration. In the eye, identification of ocular SCs first started in the 1970s. The ocular SCs are region-specific and are commonly found in the limbus, conjunctiva and trabecular meshwork [226]. Later, a clinical study investigated SC populations in peripheral blood samples from preterm babies with or without ROP [227]. Two SC populations were found to be upregulated in preterm babies and babies with ROP. They are very small embryonic-like stem cells (VSEL-SCs, Lin^−^CXCR4^+^CD45^−^) and endothelial progenitor cells (EPCs, CD34^+^CD133^+^CD144^+^), respectively. VSEL-SCs express the early embryonic transcription factors and play a role in embryogenesis. Clinical studies indicated that the mobilization of VSEL-SCs is activated in some hypoxic-related injuries, such as acute myocardial infarction and stroke, and accumulated in peripheral blood [228,229]. However, the actual role of VSEL-SCs in tissue repair is still unknown. On the other hand, EPCs are responsible for tissue repair. EPCs can differentiate into endothelial cells, and they can be transported from bone marrow to the site of injury through circulation [227]. The findings that mobilization of systemic EPCs is upregulated during normal vascular development without ROP and in the proliferative phase of ROP suggested that a specific SC mobilization is associated with ROP progression.

In fact, different populations of SCs have been shown to promote retinal vascular repair in the mouse OIR model. Adult mouse bone marrow (BM)-derived lineage negative (Lin^−^) or CD44^hi^ myeloid progenitor cells are the first SC populations that are investigated in OIR [230]. This study demonstrated that adult BM derived myeloid progenitor cells could migrate to the avascular zone in the retina and differentiate into microglia. Moreover, these SCs did not only reduce avascular and neovascular areas after injection, but also accelerated retinal revascularization. Retinal morphology and function were also examined for the long-term effect after transplantation. No abnormal retinal structure and function were observed between injected eyes and control eyes at six months after injection. Another study of SCs in OIR used two main subtypes of EPC, early EPCs (eEPCs) and outgrowth endothelial cells (OECs) [231]. Their proliferative potential was investigated in the in vitro culture. The lower proliferative property was shown in the eEPCs comparing with OECs, although both displayed the classical EPC phenotypes, such as bound lectin and expressed CD31. Furthermore, OECs have a higher expression of VEGFR-2 and promote cell monolayer formation with intercellular junctions. Although both eEPCs and OECs yielded EPC phenotypes, only OECs are closely related to endothelial cells by displaying tight junctions and association with retinal vascular formation in the in vitro study. OECs were then injected intravitreally after hyperoxia for further investigation on the role of OECs in the OIR model. Injected eyes displayed a significantly reduced avascular area and prevention of neovascularization. Other than eEPC and OEC, the effect of mesenchymal stem cells (MSCs) and adipose-derived stem cells (ASCs) were reported recently using the mouse OIR model [232,233,234,235]. MSCs or ASCs were injected into the OIR-treated mice at P12 intravitreally. The injection of MSCs did not only protect the retinal vessels by reducing avascular and neovascular areas, but also inhibited inflammatory activity. Lower expression of proinflammatory cytokines, such as IL-1β and TNFα, and increased anti-inflammatory cytokines, including IL-10 and IL-4, was observed. On the other hand, the ASC-injected pups displayed a positive effect in retinal morphology by increase the retinal vascular area after OIR. Despite its therapeutic potential in protecting vasculature and inhibiting inflammation in the OIR model, effects of SCs on retinal neuronal morphology and function are not studied and reported. Therefore, more investigations are needed for stability, efficacy and side effects of SC therapy in the developing infant eye.

## 7. Current Treatments in ROP

Current treatments for ROP aim to control and eliminate the pathological neovascularization; they include cryotherapy, laser photocoagulation and anti-VEGF therapy [236]. Retinal cryotherapy and laser coagulation involve ablation of the peripheral avascular retina [237]. Cryotherapy was first described as a treatment for severe ROP patients in the late 1980s. It is an effective treatment, yielding more than 40% reduction of unfavorable functional outcomes in the cryotherapy-treated eye of severe ROP infants in 3-month, 10-year and 15-year follow-up CRYO-ROP studies [238,239,240]. However, complications appeared with cryotherapy, such as conjunctival laceration, lid edema, apnea, and vitreous hemorrhage, as well as new retinal detachment [9,226,240]. For the better structural and functional outcomes, laser therapy emerged with less ocular and systemic side effects [9,226,241]. It has a lower requirement in general anesthesia. However, some complications about laser treatment are substantial, such as cataract, intraocular hemorrhage, and cornea and lens burn [226]. Ablative therapy has an effective outcome in ablation of peripheral avascular retina after treatment, but it may occur with systemic complications or unfavorable structural and functional outcomes.

It has been shown that upregulation of VEGF in the progression of ROP causes retinal neovascularization. Recently, extensive research has been focused on controlling VEGF concentration during hypoxia. Intravitreal injection of anti- VEGF antibody has been performed to normalize the excess VEGF. Bevacizumab, ranibizumab and aflibercept are the anti-VEGF antibodies that underwent ROP clinical trials since 2011. Reduced retinal vascular tortuosity, and in turn, regression of vessel growth toward peripheral retina were observed after injection [54,61]. The side effects that are observed laser in ablative therapy, such as retinal scarring and cataract are eliminated. However, other clinical trials reported that the injected anti-VEGF antibodies could enter the systemic circulation through the damaged BRB, and in turn, reduce the systemic VEGF amount [55,56], which may affect the development of the infants. Therefore, long-term studies in using anti-VEGF treatment for ROP infants are essential for preventing neurodevelopment disruption in their childhood.

Combination treatment of laser coagulation and anti-VEGF injection has now become a new trend of ROP treatment. Previous clinical studies in combination therapy have a positive and effective outcome for ROP patients [57,58,62,217]. Regression of peripheral retinal vessel, while no signs of ocular and systemic side effects were observed after bevacizumab injection and laser treatment during the 8-week, 3-month and 6-month follow-up period [57,58,217]. Another clinical trial using ranibizumab injection with laser therapy showed regression of ROP without any unfavorable ocular outcome [62]. In fact, the combined therapy of anti-VEGF injection and laser coagulation may require a lower anti-VEGF dose, thereby minimizing the systemic drop of VEGF level after therapy with a better outcome. However, long-term study about the efficacy and adverse effects of therapy are necessary.

## 8. Conclusions and Future Perspectives

Since 1942 when ROP was first described, many therapeutic options in inhibiting VEGF actions have been proposed and provided retinal vascular protective effects. Unfortunately, the protection of retinal neurons in OIR and ROP attracts little attention. Many antioxidants, anti-inflammatory agents and other factors could be the targets of neuroprotection. However, despite their availability, there have been very limited investigations in OIR and ROP that focused on the preservation of neuronal structure and function using these agents. Future studies should be conducted in deciphering the relationship between vascular changes and neurodegeneration in ROP and functional and morphological changes in neuronal cells, as well as glial cells, so as to preserve vision in babies with ROP, a lifelong medical condition and a major public health issue.

## Figures and Tables

**Table 1:** Vascular protective agents in the role of ROP development.

Pathogenic Agents in ROP	Phase 1 in ROP Development	Phase 2 in ROP Development	Relevant Vascular Protective Agents in ROP Development (Phase of ROP)	Intervention	Animal Model	Beneficial Effect	Adverse Effect	Reference
VEGF	↓	↑	VEGF (Phase 1)	Intraocular injection	Rat OIR model	Prevention of apoptosis of vascular endothelial cells Reduction in avascular area	/	[16]
			Bevacizumab (Phase 2)	Intravitreal injection	(Clinical study)	Reduction of the avascular area and continuing vessels growth	Reduced systemic VEGF level after injection Influenced the long-term neurodevelopment	[17,18,19,20,21,22,23]
			Ranibizumab (Phase 2)	Intravitreal injection	(Clinical study)		/	[19,24,25]
			Aflibercept (Phase 2)	Intravitreal injection	Mouse OIR model		/	[26]
			VEGFA shRNA (Phase 2)	Subretinal injection	Rat OIR model	Reduced VEGF expression Inhibited neovascularization in short- and long-term studies	/	[27]
			Anti-KDR (Phase 2)	Surgical implantation	Dog OIR model	Suppressed neovascularization	/	[28]
			SRPIN340 (Phase 2)	Intraocular injection	Rat OIR model	Reduced VEGF expression	/	[29]
			Rapamycin (Phase 2)	Subcutaneous injection	Mouse OIR model	Reduced neovascularization	/	[30]
IGF-1	↓	↑	rhIGF-1 (Phase 1)	Intraperitoneal injection	Mouse OIR model	Higher score in maturation assessments Reduced neovascularization	/	[31]
			IGFBP3 (Phase 1 and 2)	Knockout mouse	Mouse OIR model	Reduced neuronal cell apoptosis Larger retinal avascular area	/	[32,33]
			Jb3 (Phase 2)	Subcutaneous injection	Mouse OIR model	Less neovascular tufts formation	/	[34]
Epo	↓	↑	/	/	/	/	/	/
HIF-1	↓	↑	DMOG (Phase 1)	Intraperitoneal injection	Mouse OIR model	Prevented vessel loss and vessel tufts formation	/	[35]
			PHD2 (Phase 1 and 2)	Knockout mouse	Mouse OIR model		/	[36]
			RTP801 (Phase 1 and 2)	Knockout mouse	Mouse OIR model	Induced transcription of HIF-1 in phase 2 Reduced neovascular tufts and lower apoptosis	/	[37]
NO	↓	↑	l-NA (Phase 2)	Intraperitoneal injection	Rat OIR model	Reduced avascular zone and neovascular tufts	/	[38]
			l-NNA (Phase 2)	Intraperitoneal injection	Mouse OIR model		/	[39]
			AG (Phase 2)	Intravitreal injection	Mouse OIR model		/	[40]
Adenosine	↓	↑	/	/	/	/	/	/
β-AR	?	↑	Propranolol (Phase 2)	Subcutaneous injection	Mouse OIR model	Reduced neovascularization Reduced VEGF expression	/	[41]
				Topical administration	Mouse OIR model		/	[42]
			Atenolol (Phase 2)	Subcutaneous injection	Mouse OIR model	Reduced avascular zone and neovascular tufts	/	[43]
			ICI 118,551 (Phase 2)				/	
			SR59230A (Phase 2)				/	
Other angiogenic agents	?	↑	Dexamethasone (Phase 2)	Subcutaneous injection	Mouse OIR model	Suppressed neovascular formation	Steroid has a higher risk of severe ROP and fungal sepsis development	[44]
			Anecortave acetate (Phase 2)	Intravitreal injection	Rat OIR model			[45]
			Degulin (Phase 2)	Intravitreal injection	Mouse OIR model	Regulated HIF-1α suppression	/	[46]
			YC-1 (Phase 2)	Intravitreal injection	Mouse OIR model		/	[47]
			β-lapachone (Phase 2)	Intravitreal injection	Mouse OIR model		/	[48]
			16K HPRL (Phase 2)	Intravitreal injection	Mouse OIR model	Mediated endothelial cell proliferation	/	[49]
			12-LOX (Phase 2)	Intraperitoneal injection	Mouse OIR model		/	[50]
			TMP (Phase 2)	Intraperitoneal injection	Mouse OIR model	Prevented neovascular formation	/	[51]
			K5 (Phase 2)	Intravitreal injection	Rat OIR model		/	[52]
			MEF2C (Phase 1 and 2)	Knockout mouse	Mouse OIR model		/	[53]

Abbreviations: Short hairpin RNA linked VEGF (VEGFA shRNA), kinase insert domain-containing receptors (KDR), recombinant human IGF-1 (rhIGF-1), IGF binding protein (IGFBP), dimethyloxalylglycine (DMOG), prolyl hydroxylase (PHD), nitric oxide (NO), N-nitro-l-arginine (L-NA), N G-nitro-l-arginine (L-NNA), aminoguanidine (AG), N-terminal fragment of human prolactin (16K HPRL), 12-lipoxygenase (12-LOX), tetramethylpyrazine (TMP), plasminogen kringle (K5), myocyte enhancer factor 2 C (MEF2C). ↑ = upregulated; ↓ = downregulated; ? = unknown.

## Abbreviations

5′ N	5′ nucleotidase
AD	Alzheimer’s disease
AMD	Age-related macular degeneration
AT1-R	Angiotensin II type-1 receptor
β-AR	β-adrenergic receptor
BRB	Blood-retinal barrier
COX	Cyclooxygenase
DHA	Docosahexaenoic
DR	Diabetic retinopathy
EPA	Eicosapentaenoic
eEPC	Early endothelial progenitor cells
Epo	Erythropoietin
FDA	Food and Drug Administration
G-CSF	Granulocyte colony-stimulating factor
HIF	Hypoxia-inducible factor
HRE	Hypoxia response element
HSPG	Heparin sulphate proteoglycans
IGF-1	Insulin-like growth factor-1
KDR	Kinase insert domain-containing receptor
KLT-1	Kms-related tyrosine kinase 1
mTOR	Mammalian target of rapamycin
NOS	Nitric oxide synthetase
NRP	Neurophilin
ω-3 PUFAs	Omega-3 long-chain polyunsaturated fatty acids
OIR	Oxygen-induced retinopathy
OPC	Outgrowth endothelial cells
PD	Parkinson’s disease
PHD	Prolyl hydroxylase
RAS	Renin-angiotensin system
RGC	Retinal ganglion cells
ROP	Retinopathy of prematurity
ROS	Reactive oxygen species
SC	Stem cell
SRPK1	Serine arginine protein kinase 1
SRSF1	Serine-rich splicing factor-1
STOP-ROP	Supplemental Therapeutic Oxygen for Prethreshold Retinopathy Of Prematurity
VEGF	Vascular endothelial growth factor
